# The association of serum vascular endothelial growth factor levels and psoriasis vulgaris: A protocol for systematic review and meta-analysis

**DOI:** 10.1097/MD.0000000000021565

**Published:** 2020-08-14

**Authors:** Juan Gong, Hua Yang, Dongwei Qi, Xueyong Tang

**Affiliations:** Department of Dermatology, Chongqing Hospital of Traditional Chinese Medicine, Chongqing, China.

**Keywords:** protocol, psoriasis vulgaris, systematic review, vascular endothelial growth factor

## Abstract

**Background:**

In recent years, more and more attention has been paid to the role of skin microcirculation in the pathogenesis of psoriasis. The vascular network of the skin is mainly distributed in the dermis and the subcutaneous fat layer join. The microvessels are composed of terminal arterioles, arteriovenous capillaries, and postcapillary venules. Vascular endothelial growth factor (VEGF) plays an important role in the pathogenesis of psoriasis by promoting angiogenesis. The purpose of this study is to evaluate the relationship between serum VEGF and psoriasis vulgaris.

**Methods:**

Embase, CENTRAL, PubMed, China Biology Medicine Database, China National Knowledge Database, Wan Fang Database, and Chong Qing VIP Database will be searched to collect case-control studies and cohort studies and evaluate the relationship between serum VEGF and psoriasis vulgaris. The search time limits will be from the establishment of the database to December 2020. Two researchers will independently screen the studies, extract data, and evaluate the risk of bias of the studies. The Meta-analysis will be carried out with the RevMan5.3 software. The quality of all included studies will be evaluated by the Newcastle-Ottawa scale.

**Results:**

This study will evaluate the relationship between serum VEGF and the pathogenesis of psoriasis vulgaris.

**Conclusion:**

This study will provide a theoretical basis for the pathogenesis of psoriasis vulgaris.

**OSF registration number:**

DOI 10.17605/OSF.IO/6DV8P

## 1 Introduction

Psoriasis is a common, chronic, inflammatory, multisystem disease with predominantly skin and joint manifestations affecting approximately 2% of the population.^[1]^ In recent years, more and more attention has been paid to the role of skin microcirculation in the pathogenesis of psoriasis. The vascular network of the skin is mainly distributed in the dermis and the subcutaneous fat layer join. The microvessels are composed of terminal arterioles, arteriovenous capillaries, and postcapillary venules.^[2,3]^

The change of microcirculation is an important histopathological change in psoriasis, which results in structural and functional changes under the action of various pathogenic factors, resulting in the occurrence and maintenance of inflammatory reaction. The changes of microcirculation can be used as an effective index to evaluate the severity of the disease, the efficacy of drugs and the monitoring of the disease.^[2]^ Vascular endothelial growth factor (VEGF) plays an important role in the pathogenesis of psoriasis by promoting angiogenesis.^[4,5]^ Because of its complex pathogenesis, psoriasis can not be cured completely at present. The main purpose of treatment is to control and stabilize the disease, slow down the development process, reduce clinical symptoms such as erythema, scale, plaque thickening, and pruritus, reduce short-term and long-term adverse reactions, control psoriasis-related complications, and improve patients’ quality of life.^[6]^

Psoriatic lesions are characterized by a high degree of changes in the vascular network, that is, a large number of dilated, tortuous, and highly permeable skin vessels. The secretion of a variety of angiogenic growth factors promotes the expansion of the vascular network of psoriatic skin cells. Angiogenesis in psoriasis is closely related to vascular endothelial activation, angiogenesis, inflammation, and skin lesions.^[7]^ The formation of skin blood vessels includes vasculogenesis and angiogenesis,^[8]^ and vasculogenesis is a key factor in the pathogenesis of psoriasis. VEGF is the mediator of pathological angiogenesis and is overexpressed in the skin of patients with psoriasis.^[9]^ VEGF not only promotes skin angiogenesis in patients with psoriasis, but also acts as an autocrine regulator of epidermal hyperplasia,^[10]^ driving psoriasis keratin imbalance and epidermal hyperplasia,^[11]^ thus forming a characteristic lesion of psoriasis.

Besides, VEGF promotes the formation of cell adhesion molecules in capillaries and increases vascular permeability, resulting in the migration of leukocytes to the skin of patients with psoriasis. This process leads to the increase of oxygen consumption and the activation of angiogenic transcription factors, which makes it difficult to heal the angiogenesis and inflammation of psoriasis repeatedly.^[4]^ The correlation study on the mechanism of angiogenesis in psoriasis vulgaris showed that the expression of VEGF in skin lesions and serum of patients with psoriasis vulgaris was significantly higher than that in normal controls, suggesting that VEGF may play an important role in angiogenesis and inflammation of psoriasis and can be used as an index to evaluate the severity of psoriasis vulgaris.^[7]^ Related studies also show that VEGF may be an important index to judge the activity of psoriasis vulgaris.^[4,12,13]^

## 2 Methods

This protocol has been registered in the Open Science Framework. The system review will be submitted to peer-reviewed journals. This protocol will be based on the preferred reporting items for systematic reviews and meta-analysis protocols 2015 statement.^[14]^

### 2.1 Inclusion criteria

#### 2.1.1 Type of study

All case-control studies and cohort studies that evaluate the relationship between serum VEGF and psoriasis vulgaris.

#### 2.1.2 Types of participants

Patients diagnosed with psoriasis vulgaris, regardless of nationality, race, gender, age, and course of the disease.

#### 2.1.3 Exposure factors

The levels of serum VEGF.

#### 2.1.4 Outcomes

The expression levels of serum VEGF and its correlation with psoriasis vulgaris.

### 2.2 Exclusion criteria

The exclusion criteria will be as follows: duplicate literature; literature with only abstracts but no full text; literature with incomplete data; studies involving animal experiments; patients who have taken steroids within the preceding two weeks and/or taken retinoids or topical steroids within the preceding week.

### 2.3 Electronics searches

Embase, CENTRAL, PubMed, China Biology Medicine Database, China National Knowledge Database, Wan Fang Database, and Chong Qing VIP Database will be searched to collect case-control studies and cohort studies and evaluate the relationship between serum VEGF and psoriasis vulgaris. The search time limit will be from the establishment of the database to December 2020.

### 2.4 Search strategy

The keywords of the search will include ‘Vascular Endothelial Growth Factors’, ‘Vascular Endothelial Growth Factor’, ‘VEGF’, ‘Vascular Endothelial Growth Factor A’, ‘Vascular Endothelial Growth Factor B’, ‘Vascular Endothelial Growth Factor C’, ‘Vascular Endothelial Growth Factor D’; ‘psoriasis’, ‘psoriasis vulgaris’, ‘plaque psoriasis’, ‘guttate psoriasis’; ‘Case-Control Studies’, ‘Case-Control Study’, ‘Studies, Case-Control’, ‘Study, Case-Control’; ‘Cohort Studies’, ‘Cohort Study’, ‘Studies, Cohort’, ‘Study, Cohort’.

### 2.5 Data extraction and management

Two researchers will independently screen the studies, extract data, and perform crosschecks. Differences will be solved through discussion with a third researcher. The data extraction includes the first author, publication time, country, sample size, VEGF detection methods, the VEGF types, outcome measure (the expression levels of serum VEGF and its correlation with psoriasis vulgaris).

### 2.6 Data synthesis and analysis

Meta-analysis will be performed with RevMan V.5.3 software. Standardized mean difference will be used for continuous variables, and the 95% CI will be given for each effect size.

### 2.7 Assessment of heterogeneity

The studies’ heterogeneity will be assessed by using the I^2^ index. If the I^2^ < 50%, studies will not be considered heterogeneous, and the fixed-effects model will be used for meta-analysis. If the I^2^ > 50%, we will analyse the cause of the heterogeneity by using subgroup and sensitivity analysis, and the random-effects model will be used for meta-analysis.

#### 2.7.1 Subgroup analysis

If there is significant heterogeneity in the included studies, we will conduct subgroup analysis according to the study types, VEGF detection methods, and the VEGF types.

#### 2.7.2 Sensitivity analysis

Sensitivity analysis will be conducted to check the stability of the outcome by rejecting low-quality studies.

#### 2.7.3 Assessment of publication bias

The funnel plots will be used to assess publication bias.

#### 2.7.4 Quality assessment

The quality of the included study will be assessed by the Newcastle-Ottawa scale (NOS).^[15,16]^ The NOS ranges between zero up to nine stars. It will evaluate the quality of each study from the following three aspects: the selection of the study groups, the comparability of the groups, the exposure of case-control studies, and the outcome of cohort studies. The total scores are categorized into three groups: very high risk of bias (0 to 3 NOS points), high risk of bias (4 to 6), and low risk of bias (7 to 9).^[17]^

## 3 Discussion

Several previous clinical studies have reported the relationship between serum VEGF and psoriasis,^[18–20]^ but there are few systematic reviews to explore the relationship between serum VEGF and psoriasis vulgaris. In this study, we will systematically evaluate the relationship between serum VEGF and the pathogenesis of psoriasis vulgaris through a comprehensive search of the literature. The results of this study will summarize the latest evidence about the relationship between serum VEGF and psoriasis vulgaris. This study will not only provide useful evidence for patients and clinicians but also provide a theoretical basis for the pathogenesis of psoriasis vulgaris.

However, this systematic review may have several limitations. First of all, the databases we searched are limited and do not represent all the studies. Secondly, we will include both case-control studies and cohort studies, which may result in substantial heterogeneity. Third, there are a variety of methods for the detection of serum VEGF, which will become a factor in heterogeneity. We hope that this systematic review and Meta analysis can provide reliable evidence for the association between serum VEGF and psoriasis vulgaris.

## Author contributions

**Conceptualization:** Juan Gong, Dongwei Qi.

**Investigation:** Hua Yang, Xueyong Tang.

**Supervision:** Juan Gong, Dongwei Qi.

**Writing – original draft:** Hua Yang, Xueyong Tang.

**Writing – review & editing:** Hua Yang, Dongwei Qi.
